# Regulation of Imiquimod-Induced Mouse Psoriasis Development via Apoptosis Signal-Regulating Kinase 1 Potentially by Antagonizing Aryl Hydrocarbon Receptor Expression

**DOI:** 10.3390/cimb48070653

**Published:** 2026-06-25

**Authors:** Hideaki Hasegawa, Aruma Watanabe, Yasuhiro Katahira, Izuru Mizoguchi, Tatsuo Maeda, Junya Mizugami, Isao Naguro, Hidenori Ichijo, Kazutoshi Harada, Yukari Okubo, Takayuki Yoshimoto

**Affiliations:** 1 Department of Immunoregulation, Institute of Medical Science, Tokyo Medical University, 6-1-1 Shinjuku, Shinjuku-ku, Tokyo 160-8402, Japan; 2 Department of Dermatology, Tokyo Medical University, 6-7-1 Nishi-Shinjuku, Shinjuku-ku, Tokyo 160-0023, Japan; 3 Laboratory of Cell Signaling, Graduate School of Pharmaceutical Sciences, The University of Tokyo, 7-3-1 Hongo, Bunkyo-ku, Tokyo 113-0033, Japan

**Keywords:** apoptosis signal-regulating kinase 1, aryl hydrocarbon receptor, imiquimod-induced psoriasis, cytochrome P450 1A1

## Abstract

Imiquimod-induced skin inflammation is the most widely used psoriasis mouse model. Although p38 mitogen-activated protein kinase reportedly plays a role in the pathogenesis of psoriatic inflammation, the purpose of one of its upstream activators, apoptosis signal-regulating kinase 1 (ASK1), remains unclear. This study investigated the role of ASK1 and its molecular mechanism in the imiquimod-induced psoriasis model. Compared to wild-type mice, the ASK1 knockout (KO) mouse skin lesion showed a higher clinical score and a thicker epidermis. The mRNA expression of pro-inflammatory cytokines, such as IL-17 and TNF-α, was also higher. Notably, the expression of aryl hydrocarbon receptor (AhR), a sensor for xenobiotic chemicals that is expressed in the skin to strengthen the skin barrier and accelerate terminal differentiation of the epidermis—as well as its downstream molecule CYP1A1, but not NRF2—was increased in the ASK1 KO psoriatic skin lesion. Immunoprecipitation analysis, followed by Western blotting, revealed that ASK1 interacts with AhR in cells transfected with their respective expression vectors, potentially leading to reduced AhR expression. These results suggest that ASK1 negatively regulates the development of the imiquimod-induced mouse psoriasis model by interacting with AhR and presumably antagonizing the AhR-CYP1A1 axis.

## 1. Introduction

Psoriasis is a chronic inflammatory autoimmune skin disease dependent on CD4^+^ T helper (Th)17 and Th22 cells that produce interleukin (IL)-17 and IL-22, similar to rheumatoid arthritis and Crohn’s disease [1,2,3,4]. Psoriasis affects more than a million people worldwide, with an estimated prevalence of 2–4% of the population of Western countries [5]. Psoriasis is characterized by inhibiting the terminal differentiation of epidermal keratinocytes and promoting their proliferation through cross-talk between immune cells and skin cells, as well as through oxidative stress [6]. This leads to reduced expression of filaggrin and loricrin, which are responsible for the skin’s barrier function [7]. Although the pathogenesis is complicated, it is thought that factors, such as environmental stressors—both internal and external—climate change, underlying constitutional predispositions, mental and physical stress, and insufficient ultraviolet exposure, may be involved [8].

It was previously reported [9] that frequent application of Beselna cream (a 5% ointment containing imiquimod, a Toll-like receptor 7 ligand) induces psoriasis-like skin symptoms. Since then, its application has been widely used as a method of modeling psoriasis. Psoriasis onset involves inflammation and stress induced via Beselna cream application, leading to cell death in keratinocytes. This produces inflammatory cytokines, such as interferon (IFN)-α/β, from plasmacytoid dendritic cells following the promotion of myeloid DC maturation, and subsequently produces IL-12 and IL-23 [10,11]. This then differentiates naïve CD4^+^ T cells into Th1 and Th17 cells secreting tumor necrosis factor (TNF)-α, IL-17 and IL-22. IL-23 also induces IL-17 and IL-22 production in γδ T cells. Keratinocytes are then activated by these cytokines and produce antimicrobial peptides, chemokines and cytokines. This process leads to the infiltration of inflammatory cells, such as neutrophils and macrophages. In particular, IL-22 induces keratinocyte proliferation and inhibits terminal differentiation, thereby accelerating keratinocyte turnover and inducing the characteristic symptoms of psoriasis [12]. Consistent with this, frequent intradermal administration of IL-22 protein has reportedly induced psoriasis-like inflammation [13].

Mitogen-activated protein kinase (MAPK) is a highly conserved intracellular signaling pathway found in organisms ranging from yeast to more complex plants and mammals [14,15]. It is involved in regulating numerous cellular functions, including proliferation, differentiation, cell death and stress responses. Previous studies have implicated environmental factors, such as external stress, as triggers for psoriasis vulgaris. Studies indicate that the stress-responsive MAPK p38 is involved in the disease process, with the activation of p38 and ERK being observed at the onset of psoriasis [15,16]. Furthermore, there are reports showing that inhibiting p38 activity in a psoriatic skin graft model suppresses lesion progression [17]. More recently, it has been reported that p38 activator anisomycin exacerbates skin symptoms in the IMQ-induced psoriasis model, while p38 inhibitor BIRB796 alleviates them [18], and p38α-deficiency ameliorates psoriasis development by downregulating STAT3-mediated keratinocyte proliferation and cytokine production [19].

Apoptosis signal-regulating kinase-1 (ASK1), one of the MAP kinase kinase kinases (MAP3Ks), is a stress response molecule that functions upstream of the c-Jun N-terminal kinase (JNK) and p38 MAP kinase pathways in response to various environmental changes. It is activated in a stimulus- and cell-type-specific manner [20,21]. ASK1 was identified as a molecule activated by TNF-α stimulation that induces cell death [22]. It is also activated by reactive oxygen species (ROS) and Ca^2+^ and functions as a signaling pathway that controls cell survival, proliferation and differentiation in response to various environmental stresses. Analysis of ASK1 knockout (KO) mice has revealed that ASK1 is an essential signaling molecule for cell death induced by oxidative and endoplasmic reticulum stresses [23]. ASK1 also plays a crucial role in the innate immune response by selectively activating p38 and JUN downstream of TLR. Furthermore, it is known that, in psoriasis patients, the granular layer of the epidermis gradually disappears as the disease develops [24]. ASK1 expression in keratinocytes specifically suppresses proliferation and increases differentiation marker expression. Conversely, inducing keratinocyte differentiation with Ca^2+^ increases ASK1 expression, suggesting that ASK1 acts as a differentiation-inducing factor for keratinocytes [24,25].

The aryl hydrocarbon receptor (AhR) is also a key factor in the differentiation of skin keratinocytes. AhR is a ligand-activated transcription factor that recognizes chemical substances such as dioxins found in air pollutants and skin flora, as well as 6-formylindolo(3,2-b)carbazole (FICZ) [26]. FICZ is a metabolite of tryptophan that is produced by ultraviolet irradiation and induces enzymes that metabolize these substances. In CD4^+^ T cells, AhR induces Th17 and Th22 cell differentiation, as well as IL-17 and IL-22 production [27]. In keratinocytes, AhR enhances the expression of filaggrin and other genes to maintain final differentiation and skin barrier function [28]. Furthermore, AhR activates the transcription factor nuclear factor erythroid 2-related factor 2 (NRF2), which protects cells from oxidative stress by suppressing ROS production and maintaining skin homeostasis. Conversely, it activates the cytochrome P450 1A1 (CYP1A1) drug-metabolizing enzyme, thereby inducing drug detoxification and ROS production. Increased AhR expression has been reported in psoriasis patients [29,30], and depending on the type of ligand, AhR activates either the NRF2 [31] or CYP1A1 [32] pathway. This results in complex and differing contributions to the disease state [28]. AhR ligands such as FICZ [33] and tapinarof [34,35] have been reported to reduce psoriasis symptoms via NRF2, whereas dioxin exacerbates symptoms via CYP1A1, IL-17 and IL-22 [36].

Therefore, in this study, we investigated the role of ASK1 and its mechanism of action in the onset and pathogenesis of IMQ-induced psoriasis using ASK1 KO mice. However, contrary to our expectations based on the results demonstrating the therapeutic effects of the p38 inhibitor against IMQ-induced psoriasis [18], the symptoms of psoriasis in ASK1 KO mice worsened. Our findings revealed that ASK1 negatively regulates the development of IMQ-induced psoriasis, potentially by antagonizing the AhR-CYP1A1 axis.

## 2. Materials and Methods

### 2.1. Mice

C57BL/6 female mice, 6–7 weeks old, were purchased from Sankyo Labo Service (Hamamatsu, Japan). ASK1 KO mice (C57BL/6 background) and control wild-type (WT) C57BL/6 mice were bred and maintained under specific pathogen-free conditions at the animal facility of Tokyo Medical University. All animal experiments were approved by the President and by the Institutional Animal Care and Use Committee of Tokyo Medical University (Approval numbers: H28003, H290069, H300102, H310042, R2-0012, R3-0064, R4-108, R5-106, R6-019 and R7-022) and performed in accordance with institutional, science community, and national guidelines for animal experimentation and the Animal Research: Reporting of In Vivo Experiments guide 2.0.

### 2.2. IMQ-Induced Psoriasis Model Mouse

This model mouse was established as described previously [9]. Briefly, the back skin of mice was shaved using an electric clipper and then treated with a depilatory cream to remove residual hairs 2 days before treatment. ASK1 KO mice and WT mice (*n* = 3–5) were untreated or topically treated with daily administration of 62.5 mg of 5% IMQ cream (Beselna cream provided by Mochida Pharmaceutical, Tokyo, Japan) or Vaseline as a control cream on the shaved back skin for 4–6 consecutive days [9]. Sample sizes were determined based on previous studies and common practice in the field to ensure sufficient statistical robustness. The mice were randomly allocated to each group. Mice were excluded if they developed an unrelated illness prior to IMQ treatment. During this period, the skin was observed daily, and the degree of erythema, thickening, and scaling was evaluated using the scores: 0, absent; 1, mild; 2, moderate; 3, severe; and 4, very severe. Although the scores were not assessed completely blindly, the objective scoring criteria were meticulously applied to minimize personal bias.

### 2.3. Histopathological Evaluation

Mice were euthanized by cervical dislocation before dissection of tissues. The skin tissues were collected, immersed in a 10% buffered formalin solution overnight, embedded in paraffin and sectioned using a microtome. The resulting sections were stained with hematoxylin and eosin (H&E) to observe the degree of cellular infiltration, and epidermal thickness was quantified using an image analysis software (Fiji, an expanded version of ImageJ, version 1.53c; National Institutes of Health, Bethesda, MD, USA) after capturing images. Additionally, skin tissue treated with IMQ was embedded in OCT compound and frozen, and the frozen sections were prepared using a microtome. After sectioning, the sections were blocked, then stained with anti-CYP1A1 (Clone # B-4, Santa Cruz, Dallas, TX, USA) or anti-NRF2 (Clone # H-300, Santa Cruz), and detected using the VECTASTAIN Elite ABC Kit (Vector, Newark, CA, USA) together with counterstaining using H&E. Quantification of the intensity of immunostaining was conducted with Fiji software.

### 2.4. Reverse Transcription–Quantitative Polymerase Chain Reaction (RT-qPCR)

Total RNA was extracted from the skin tissues using RNeasy Mini Kit (Qiagen, Venlo, The Netherlands), and cDNA was prepared using oligo(dT) primer and SuperScript IV RT (Invitrogen, Waltham, MA, USA). Real-time quantitative PCR was performed using KAPA SYBR Fast qPCR Master Mix (Kapa Biosystems, Wilmington, MA, USA) and a Thermal cycler Dice real-time system (Takara, Shiga, Japan) according to the manufacturer’s instructions. Hypoxanthine phosphoribosyl transferase (HPRT) was used as a housekeeping gene to normalize mRNA. Relative expression of qPCR products was determined by using the ΔΔCt method to compare target gene and housekeeping gene mRNA expression. The specific primer pairs used were as follows: TNF-α, 5′-TATGGCCCAGACCCTCACA-3′ and 5′-GGAGTAGACAAGGTACAACCCATC-3′; IFN-γ, 5′-CGGCACAGTCATTGAAAGCCTA-3′ and 5′-GTTGCTGATGGCCTGATTGTC-3′; IL-17A, 5′-CTGATCAGGACGCGCAAAC-3′ and 5′-TCGCTGCTGCCTTCACTGTA-3′; IL-1β, 5′-TCCAGGATGAGGACATGAGCAC-3′ and 5′-GAACGTCACACACCAGCAGGTTA-3′; IL-22, 5′-TTCCAGCAGCCATACATCGTC-3′ and 5′-CTTCCAGGGTGAAGTTGAGCA-3′; HPRT, 5′-TTGTTGTTGGATATGCCCTTGACTA-3′ and 5′- AGGCAGATGGCCACAGGACTA-3′.

### 2.5. Western Blotting

Skin tissues were minced in a RIPA lysis buffer (50 mM Tris-HCl pH7.6 containing 150 mM NaCl, 1% Triton X-100, 1% sodium deoxycholate, and 0.1% SDS) containing a protease inhibitor cocktail, left on ice, sonicated, and then centrifuged at 4 °C. The resultant cell lysates were separated by SDS-PAGE under reducing conditions and transferred to an Immobilon membrane (Merck Millipore, Burlington, MA, USA). After blocking the membrane, it was incubated with the following antibodies: ASK1 (Clone # EP553Y, Abcam, Cambridge, UK), AhR (Clone # W16012A, BioLegend, San Diego, CA, USA; Clone # A-3, Santa Cruz), loricrin (Clone # 905104, BioLegend), involucrin (Clone # SY5, Santa Cruz), CYP1A1 (Clone # B-4), NRF2 (Clone # H-300), HA (Clone # 6E2, Cell Signaling, Danvers, MA, USA; Clone # Y11, Santa Cruz), c-MYC (Clone # 9E10, Santa Cruz), and β-actin (Clone # C4, Santa Cruz), and incubated overnight at 4 °C. Subsequently, each appropriate secondary antibody conjugated with horseradish peroxidase was applied, and the luminescent signal was detected using the Amersham ECL Prime Western Blotting Detection Reagent (Cytiva, Tokyo, Japan) on an iBright FL 1500 imaging system (Thermo Fisher Scientific, Waltham, MA, USA). Quantification of the band intensity was conducted with Fiji software.

### 2.6. Cell Culture

Human embryonic kidney cell line HEK293T (from J. Mizuguchi) and human hepatocellular carcinoma cell line HepG2 (from J. Miyazaki) were cultured in DMEM (Invitrogen) containing 10% fetal bovine serum, 100 U/mL penicillin and 100 μg/mL streptomycin (Invitrogen). Mouse keratinocyte cell line PAM212 [37] (from Dr. Tajima) was cultured in RPMI1640 (Sigma-Aldrich, St. Louis, MA, USA) containing 10% fetal bovine serum, 50 μM 2-mercaptoethanol, 100 U/mL penicillin and 100 μg/mL streptomycin (Invitrogen).

### 2.7. Plasmids

As an ASK1 plasmid, pcDNA3-HA-tagged mouse (m)ASK1 WT [22] with an N-terminal HA tag was used. As an AhR plasmid, pCMV3-mAhR-MYC with a C-terminal MYC tag was purchased from Sino Biological (Hong Kong, China).

### 2.8. Immunoprecipitation

HEK293T cells, HepG2 cells, and PAM212 cells were transfected with pcDNA3-HA-mASK1 and/or pCMV3-mAhR-MYC using FuGENE 6 (Promega, Madison, WI, USA), according to the manufacturer’s instructions. The total amount of plasmid used for transfection was adjusted to 1 μg by adding a control empty vector. After 48–72 h, cells were lysed with 1% Nonidet P-40 lysis buffer (10 mM Tris-HCl, pH 7.5, 150 mM NaCl, 1 mM EDTA) containing a protease inhibitor cocktail, followed by centrifugation. The supernatant was incubated overnight at 4 °C with anti-MYC (Clone # 9E10) or anti-HA (Clone # Y11), anti-AhR (Clone # W16012A) bound to protein G-sepharose (GE Healthcare, Chicago, IL, USA). After washing the beads, the complexes were separated by SDS-PAGE under reducing conditions and analyzed via Western blot using anti-HA (Clone # 6E2) or biotinylated anti-MYC (Clone # 9E10B) and anti-AhR (Clone # W16012A). Subsequently, each secondary antibody conjugated with horseradish peroxidase was applied, and the luminescent signal was detected using the Amersham ECL Prime Western Blotting Detection Reagent on an iBright FL 1500 imaging system (Thermo Fisher Scientific, Waltham, MA, USA). Quantification of the band intensity was conducted with Fiji software.

### 2.9. Statistical Analysis

Data are expressed as the mean ± standard deviation (SD) of the mean for each group. Statistical analyses were performed using the unpaired, two-tailed Student’s *t*-test for comparisons of two groups and one-way or two-way analysis of variance with Tukey’s or Dunnett’s multiple comparisons test for comparing more than three groups using GraphPad Prism 10 (GraphPad Software, San Diego, CA, USA). Repeated measures ANOVA was performed without assuming sphericity, and the Greenhouse–Geisser correction was applied where appropriate. *p* < 0.05 was considered statistically significant.

## 3. Results

### 3.1. Exacerbated Pathology of the Psoriatic Skin Lesion of ASK1 KO Mice Compared with WT Mice After IMQ Treatment

**Figure 1:**
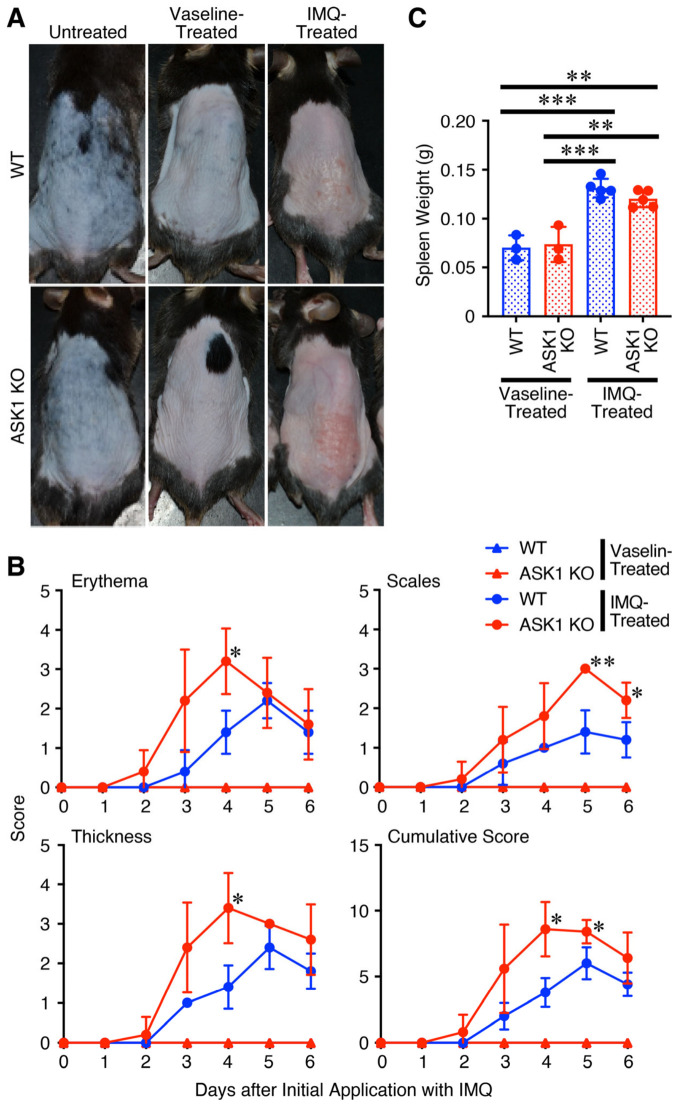
Exacerbated pathology of the psoriatic skin lesion of ASK1 KO mice compared with WT mice after IMQ treatment. ASK1 KO mice and WT mice were treated with IMQ cream (*n* = 5) or a control cream, Vaseline (*n* = 3), daily for 6 days, along with an untreated group (*n* = 2). Representative photographs of the back skin taken on day 4 are shown (**A**). Clinical scores for erythema, scales and thickness, together with their cumulative score, were measured daily during this period (**B**). Spleen weight was also measured on day 6 (**C**). Data are shown as the mean ± SD and are representative of three independent experiments. *p* values were determined using two-way analysis of variance with Tukey’s multiple comparisons test. Repeated measures ANOVA was conducted without assuming sphericity, and *p* values were corrected using the Greenhouse–Geisser method (**B**). * *p* < 0.05, ** *p* < 0.01, *** *p* < 0.001.

To investigate the role of ASK1 in the development of IMQ-induced psoriasis, WT mice and ASK1 KO mice were topically treated with IMQ cream or a control cream (Vaseline) on their shaved backs for 6 consecutive days. Clinical symptoms were observed and scored. Contrary to expectation, clinical symptoms worsened in ASK1 KO mice compared to WT mice, as seen in the dorsal skin photographs on day 4 (Figure 1A). In the skin of WT mice, clinical scores for erythema, hyperplasia, and scaling began increasing after day 3 and peaked at day 5 (Figure 1B). However, the onset of clinical scores was slightly earlier and their severity was significantly greater in the ASK1 KO mouse skin than in WT mice. Peaks in erythema and hyperplasia occurred at day 4 and cumulative scores also showed a marked difference. Moreover, application of IMQ significantly increased spleen weight, indicating splenomegaly. However, no apparent difference in the weight was observed between WT and ASK1 KO mice (Figure 1C).

These results suggest that the clinical symptoms of IMQ-induced psoriasis in the skin were actually exacerbated in ASK1 KO mice compared to WT mice, although the degree of splenomegaly remained unchanged.

### 3.2. Increased Inflammation in the Psoriatic Skin Lesions of ASK1 KO Mice Compared with WT Mice After IMQ Treatment

**Figure 2:**
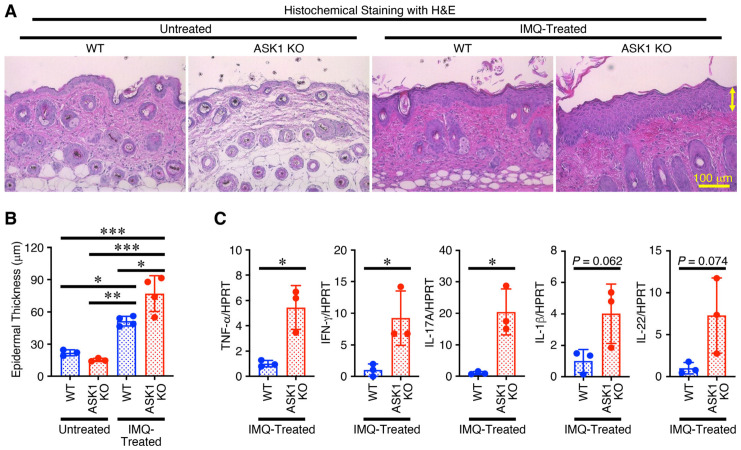
Increased inflammation in the psoriatic skin lesion of ASK1 KO mice compared with WT mice after IMQ treatment. ASK1 KO mice and WT mice were untreated (*n* = 3) or treated (*n* = 4) with IMQ cream daily for 5 days, and histochemical analysis with HE staining of the skin sections taken on day 5 was performed, and representative images are shown (**A**). The epidermal thickness (indicated by yellow double-ended arrow) of the individual skin was then measured (**B**). Data are shown as the mean ± SD and are representative of three independent experiments. On day 4, total RNA was then extracted from the skin section and subjected to RT-qPCR analysis (**C**). The expression levels of individual inflammatory cytokines were normalized to HPRT and expressed as fold changes relative to WT (*n* = 3). Data are shown as the mean ± SD and are representative of two independent experiments. *p* values were determined using two-way analysis of variance with Tukey’s multiple comparisons test (**B**) and unpaired, two-tailed Student’s *t*-test (**C**). * *p* < 0.05, ** *p* < 0.01, *** *p* < 0.001.

Next, the histochemical analysis of the skin obtained on day 5 was performed. Compared to WT mice, ASK1 KO mice showed increased lymphocyte infiltration and thicker epidermal layers in the skin based on the results of HE staining (Figure 2A). Indeed, when the average epidermal thickness was measured, it was found that the skin of ASK1 KO mice significantly increased in thickness compared to WT mice (Figure 2B). RNA was extracted from the skin tissue on day 4 and the mRNA expression of inflammatory cytokines was examined by RT-qPCR. Compared to WT mice, the results showed that the expression of inflammatory cytokines such as TNF-α, IFN-γ, IL-17, IL-1β and IL-22 was higher in the skin of ASK1 KO mice (Figure 2C).

The above results show that skin inflammation symptoms induced by IMQ were exacerbated in ASK1 KO mice compared to WT mice. Consistent with this, there was increased lymphocyte infiltration into the skin, greater epidermal thickening, and enhanced expression of inflammatory cytokines in the ASK1 KO psoriatic skin lesion.

### 3.3. Enhanced Expression of AhR and CYP1A1 but Not NRF2 in the Psoriatic Skin Lesion of ASK1 KO Mice Compared with WT Mice After IMQ Treatment

**Figure 3:**
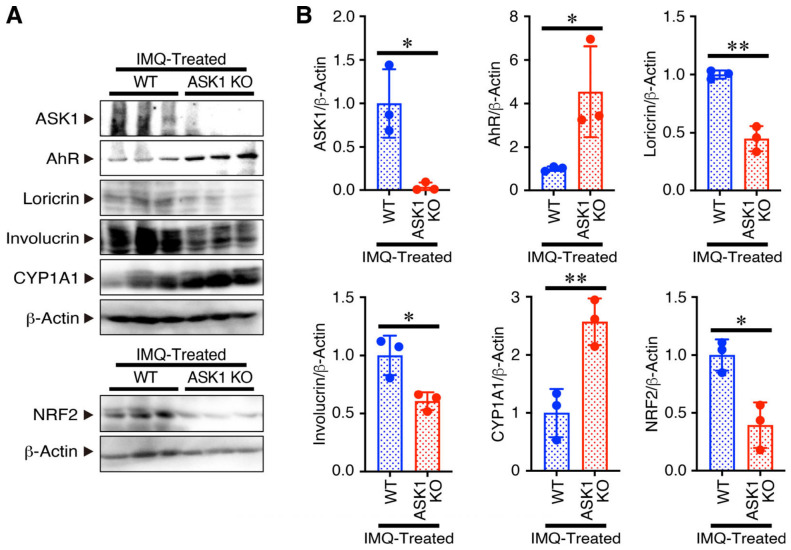
Higher expression of AhR and CYP1A1, but lower expression of NRF2, in the psoriatic skin lesion of ASK1 KO mice compared with WT mice after IMQ treatment. ASK1 KO mice and WT mice (*n* = 3) were treated daily with IMQ cream for 4 days, and cell lysates from the skin psoriasis sections were prepared on day 4 and subjected to Western blot analysis. Representative images are shown (**A**). Individual band intensities were measured using ImageJ software, normalized to β-actin, and expressed as fold changes relative to WT (**B**). Data are shown as the mean ± SD and are representative of two independent experiments. *p* values were determined using unpaired, two-tailed Student’s *t*-test. * *p* < 0.05, ** *p* < 0.01. Full-length blots are shown in Figure S3.

To investigate the mechanism of action of ASK1, cell lysates were prepared from the skin obtained on day 4 and subjected to Western blot analysis using antibodies against molecules related to skin homeostasis (Figure 3A). Notably, the expression of AhR, which acts as a sensor for xenobiotic chemicals and is abundantly expressed in the skin to strengthen the skin barrier and accelerate epidermal terminal differentiation [26], was significantly higher in the skin of ASK1 KO mice than in WT mice (Figure 3B). Preliminary data suggest that, under steady-state conditions, there is no obvious difference in AhR expression in the skin between WT and ASK1 KO mice (Figure S1). In contrast, the expression of loricrin and involucrin, which are late differentiation markers for keratinocytes expressed in the granular layer and spinous layers, respectively, decreased (Figure 3A,B). Since AhR exerts its biological activities by activating either CYP1A1 or NRF2, which lead to opposite outcomes [28], we next examined the expression of these downstream molecules in the skin. In the skin of ASK1 KO mice with IMQ-induced psoriasis, CYP1A1 expression was significantly increased compared to WT mice, while NRF2 expression was significantly decreased (Figure 3A,B).

**Figure 4:**
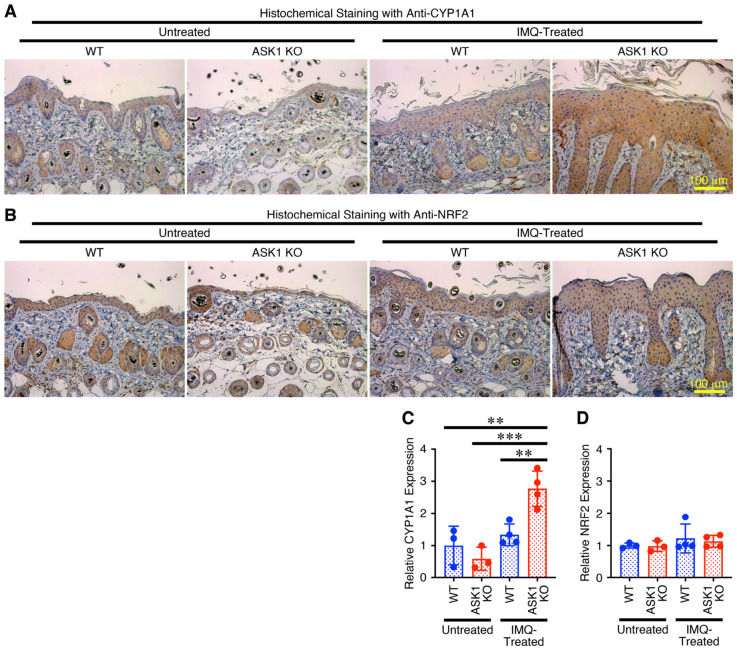
Enhanced expression of CYP1A1 but not NRF2 in the psoriatic skin lesion in ASK1 KO mice compared with WT mice after IMQ treatment. ASK1 KO mice and WT mice were untreated (*n* = 3) or treated with IMQ cream (*n* = 4) daily for 5 days. On day 5, an immunohistochemical analysis of the skin sections with antibodies against CYP1A1 (**A**) and NRF2 (**B**) was performed, and representative images are shown. The relative intensity of CYP1A1 (**C**) and NRF2 (**D**) expression was measured using Fiji software and expressed as fold changes relative to WT. Data are shown as the mean ± SD and are representative of two independent experiments. *p* values were determined using two-way analysis of variance with Tukey’s multiple comparisons test. ** *p* < 0.01, *** *p* < 0.001.

To further confirm these results, we then performed the immunohistochemical analysis of the skin obtained on day 5 using antibodies against CYP1A1 and NRF2 (Figure 4A,B). Consistent with the results obtained from Western blot analysis (Figure 3A,B), increased expression of CYP1A1 but not NRF2 was observed (Figure 4C,D).

These results suggest that, following IMQ treatment, ASK1 KO mice showed enhanced expression of AhR and CYP1A1, but not NRF2, in the psoriatic skin lesion.

### 3.4. Interaction of ASK1 with AhR in the Cells Transfected with Their Expression Vectors

**Figure 5:**
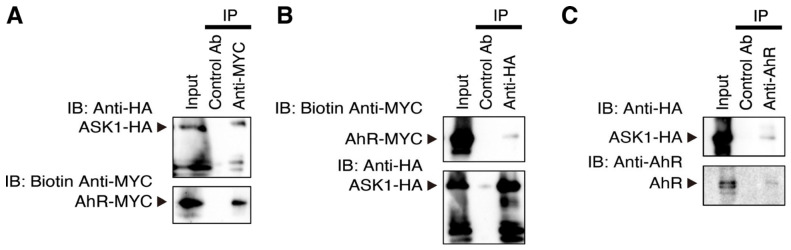
Interaction of ASK1 with AhR in the cells transfected with their expression vectors. HEK293T cells were transiently transfected with expression vectors for ASK1 and AhR, tagged with HA and MYC, respectively. Seventy-two hours later, cell lysates were prepared and immunoprecipitated with anti-MYC (**A**) or anti-HA (**B**), followed by immunoblotting (IB) with anti-HA or biotin anti-MYC, respectively. The immunoprecipitation (IP) was confirmed by detecting the immunoprecipitated proteins using the antibody that was used for the immunoprecipitation. HepG2 cells were also transiently transfected with expression vector for ASK1 tagged with HA. Seventy-two hours later, cell lysates were prepared and immunoprecipitated with anti-HA (**C**), followed by immunoblotting with anti-AhR. Data are representative of three independent experiments. Full-length blots are shown in Figure S4A (**A**,**B**) and Figure S4B (**C**).

To investigate the mechanism by which AhR expression is increased in ASK1 KO mice, we next examined the possibility of an interaction between the two. We forcibly expressed vectors carrying different tags for each protein in HEK293T cells. Using immunoprecipitation and immunoblotting analysis, we found that the two proteins co-precipitated using respective antibodies against both tags (Figure 5A,B). Moreover, HepG2 cells, which highly express endogenous AhR [38], were similarly transfected with only the ASK1-HA expression vector. The resulting cell lysates were immunoprecipitated with anti-HA, followed by immunoblotting with anti-AhR. Endogenous AhR was co-immunoprecipitated with the forcibly expressed ASK1-HA (Figure 5C).

These results suggest that ASK1 interacts with AhR, which could account for the elevated AhR expression observed in ASK1 KO psoriatic skin.

## 4. Discussion

Compared to WT mice, IMQ-induced psoriatic skin symptoms worsened in ASK1 KO mice, showing epidermal thickening and enhanced expression of inflammatory cytokines such as TNF-α, IFN-γ, IL-17, IL-1β and IL-22. Interestingly, AhR expression increased in the psoriatic skin lesion of ASK1 KO mice following IMQ treatment, while the expression of late keratinocyte differentiation markers, such as loricrin and involucrin, decreased. Furthermore, CYP1A1 expression increased in the psoriatic skin lesion of ASK1 KO mice, but NRF2 expression did not, possibly contributing to exacerbated psoriasis. One possible mechanism by which ASK1 reduces AhR expression is through its interaction with AhR. Our preliminary data from transfection experiments using increasing amounts of an HA-tagged ASK1 expression vector in the mouse keratinocyte cell line PAM212 suggest that ASK1 overexpression tends to reduce the expression of endogenous AhR (Figure S2), although further studies are needed to confirm this finding. Overall, these results suggest that ASK1 negatively regulates the development of IMQ-induced mouse psoriasis, potentially by acting as an antagonist of the AhR-CYP1A1 axis.

Previous studies have shown that ASK1 knockout mice exhibit prolonged keratinocyte activation in the wound epidermis, delayed epithelial barrier restoration, and enhanced auricular cartilage regeneration following full-thickness ear punch [39]. Similar regenerative effects were observed with topical inhibition of ASK1, its upstream activator (CaMKII), or its downstream effector (JNK), whereas inhibition of p38α/β with SB203580 had no effect [39]. These findings suggest that ASK1 suppresses keratinocyte regeneration by promoting differentiation. ASK1 activates JNK and/or p38 MAPK signaling pathways [40], both of which are implicated in keratinocyte terminal differentiation [41,42]. However, only JNK inhibition enhanced regeneration, while p38α/β inhibition did not [39]. In contrast, p38 signaling has been linked to psoriasis pathogenesis: activation by anisomycin worsens skin symptoms, whereas inhibition by BIRB796 or p38α deficiency alleviates them [18,19]. These discrepancies may reflect isoform specificity, as current inhibitors mainly target p38α/β but not p38δ [43], which plays a key role in keratinocyte function [44,45]. Additionally, transcriptomic analyses revealed that JNK inhibition promotes keratinocyte differentiation, suggesting that JNK activation may delay cornification and enhance wound healing [46]. Collectively, ASK1 and its downstream pathways have complex, context-dependent roles in psoriasis. ASK1 may suppress keratinocyte regeneration while promoting terminal differentiation, potentially via JNK and/or p38δ rather than p38α/β. Further studies are needed to clarify this mechanism.

Both ASK1 and AhR have similar effects, such as promoting keratinocyte differentiation and enhancing the differentiation into Th17 and Th22 cells. However, the present results suggest that ASK1 interacts with AhR, thereby possibly reducing AhR expression and its function. In ASK1 KO skin, AhR expression increased following IMQ treatment, as did its downstream molecule CYP1A1, but not NRF2, thereby exacerbating psoriasis. This implies that the actions and mechanisms mediated by these proteins are regulated in a more complex manner. In the human psoriasis lesion tissue, increased expression of AhR, as well as CYP1A1, was observed compared to the healthy human skin tissue [29,30]. AhR is a ligand-activated transcription factor that activates the CYP1A1 pathway in a ligand-dependent manner, exacerbating the onset of psoriasis by enhancing oxidative stress and inflammatory responses as in the case of dioxin [28,47]. In contrast, AhR also activates the NRF2 pathway, alleviating and improving psoriasis onset, as observed with FICZ and tapinarof [28].

Activation of CYP1A1 by AhR occurs directly through its function as a transcription factor, whereas NRF2 activation depends largely on stress-activated MAPK pathways, particularly JNK and p38 [48,49]. Ligand-activated AhR translocates into the nucleus, forms a heterodimer with AhR nuclear translocator, and binds to xenobiotic response elements in the CYP1A1 promoter to induce its expression [50]. In contrast, NRF2 activity is regulated by JNK and p38 through modulation of its phosphorylation, nuclear translocation, and stability [51,52]. Under basal conditions, NRF2 is sequestered by Kelch-like ECH-associated protein 1, which promotes its ubiquitination and proteasomal degradation [53]. Oxidative stress disrupts this interaction, allowing NRF2 stabilization and accumulation, with JNK and p38 further enhancing its activation. As ASK1 functions upstream of JNK and p38 [54], its knockout may attenuate NRF2 signaling and shift the response toward CYP1A1 activation. Although this scenario may help explain the underlying mechanism involving ASK1, further studies are required to confirm this.

While clinical scores commonly reach a plateau, the data for IMQ-treated ASK1-KO mice showed a peak around day 4, which occurred slightly earlier than in IMQ-treated WT mice (Figure 1B). This might be explained by the mechanism regarding the distinct temporal kinetics of CYP1A1 and NRF2 activation. This is because AhR activation, as indicated by CYP1A1 induction, represents a rapid early response, whereas NRF2-mediated antioxidant signaling, which is activated in a ROS-dependent manner and involves stress-activated MAPK pathways, particularly JNK and p38 [48,49], is likely to predominate at later stages [55]. In addition, ASK1 deficiency attenuates sustained activation of JNK and p38 MAPKs following stress stimulation, although transient early-phase phosphorylation may still occur via compensatory MAP3Ks [56]. Therefore, evaluating the temporal kinetics of AhR, JNK, p38, CYP1A1, and NRF2 in WT and ASK1 KO mice following IMQ treatment is important; however, this was not addressed in the current study and thus represents a limitation.

We observed that skin lesions in ASK1 knockout (KO) mice exhibit increased production of inflammatory cytokines, including IL-17 and IL-22 (Figure 2C), which are also known to be regulated by AhR in T cells [27]. Similarly to the skin lesions observed in ASK1 KO mice, upregulation of AhR expression in T cells would be expected to enhance the production of IL-17 and IL-22, thereby further exacerbating psoriasis. However, additional studies are needed to clarify the role of ASK1 in AhR-mediated upregulation of these cytokines in T cells.

The possible mechanisms by which ASK1 reduces AhR expression include inhibiting mRNA expression at the transcriptional level or promoting protein degradation at the protein level through proteasome- and lysosome-mediated proteolysis. However, it is more likely that ASK1 and AhR promote protein degradation via the proteasome system. This is because, interestingly, ASK1 is known to be inactivated by degradation via the ubiquitin proteasome system; however, it is reportedly associated with the deubiquitinating enzyme USP9X, which removes the ubiquitin chain from ASK1 and thereby induces the stabilization of active ASK1 [57]. If the binding of AhR to ASK1 inhibits USP9X binding to ASK1 and protects ASK1 from deubiquitination, it is conceivable that ASK1 bound to AhR could undergo enhanced degradation via the ubiquitin proteasome system. However, further studies are necessary to more precisely clarify how ASK1 interacts with AhR, reduces its expression, and regulates the development of IMQ-induced psoriasis.

## 5. Conclusions

In this study, we investigated the role of ASK1 in the pathogenesis of psoriasis and its possible mechanism of action, using an IMQ-induced psoriasis model in ASK1 KO mice. Contrary to previous findings concerning p38 MAPK, one of the downstream signaling molecules of ASK1, we observed an exacerbation of psoriatic symptoms in ASK1 KO mice. Our findings suggest that ASK1 may negatively regulate the development of IMQ-induced psoriasis, potentially by interacting with AhR to suppress its expression, thereby antagonizing the AhR-CYP1A1 axis.

## Data Availability

All data generated or analyzed during this study are included in this article.

## Supplementary Materials

The following supporting information can be downloaded at: https://www.mdpi.com/article/10.3390/cimb48070653/s1.

## Abbreviations

The following abbreviations are used in this manuscript:

AhR	aryl hydrocarbon receptor
ASK1	apoptosis signal-regulating kinase 1
CYP1A1	cytochrome P450 1A1
FICZ	6-formylindolo(3,2-b)carbazole
H&E	hematoxylin and eosin
HPRT	hypoxanthine phosphoribosyl transferase
IB	immunoblotting
IFN	interferon
IL	interleukin
IMQ	imiquimod
IP	immunoprecipitation
JNK	c-Jun N-terminal kinase
KO	knockout
MAPK	mitogen-activated protein kinase
MAP3K	MAP kinase kinase kinase
m	mouse
NRF2	nuclear factor erythroid 2-related factor 2
ROS	reactive oxygen species
SD	standard deviation
Th	T helper
TNF	tumor necrosis factor
WT	wild-type
