# Supplementary material for: Regulation of Imiquimod-Induced Mouse Psoriasis Development via Apoptosis Signal-Regulating Kinase 1 Potentially by Antagonizing Aryl Hydrocarbon Receptor Expression
Source: Curr Issues Mol Biol. 2026 Jun 25;48(7):653. doi: 10.3390/cimb48070653 (PMC13406768; doi:10.3390/cimb48070653)
Supplement: Supplementary file 1 [file cimb-48-00653-s001.zip › cimb-4367344-supplementary.pdf]

Supplementary Material

Regulation of Imiquimod-Induced Mouse Psoriasis Development via Apoptosis Signal-Regulating Kinase 1 Potentially by Antagonizing Aryl Hydrocarbon Receptor Expression

Hideaki Hasegawa [1], Aruma Watanabe [1], Yasuhiro Katahira [1], Izuru Mizoguchi [2], Tatsuo Maeda[2], Junya Mizugami [2], Isao Naguro [3,†], Hidenori Ichijo [3,‡], Kazutoshi Harada [2], Yukari Ohkubo [2] and Takayuki Yoshimoto [1,*]

[1] Department of Immunoregulation, Institute of Medical Science, Tokyo Medical University, 6-1-1 Shinjuku, Shinjuku-ku, Tokyo 160-8402, Japan
[2] Department of Dermatology, Tokyo Medical University, 6-7-1 Nishi-Shinjuku, Shinjuku-ku, Tokyo 160-0023, Japan
[3] Laboratory of Cell Signaling, Graduate School of Pharmaceutical Sciences, The University of Tokyo, 7-3-1 Hongo, Bunkyo-ku, Tokyo 113-0033, Japan
* Correspondence: yoshimot@tokyo-med.ac.jp; Tel.: +81-333516141
† Current address: Laboratory of Bioresponse Signaling, Graduate School of Pharmacy and Pharmaceutical Sciences, Juntendo University, 6-8-1 Hinode, Urayasu, Chiba 279-0013, Japan
‡ Current address: Cell Signaling and Stress Responses Laboratory, Advanced Research Departments, Institute of Science Tokyo, 2-3-10 Surugadai Kanda, Chiyoda-ku, Tokyo 101-0062, Japan

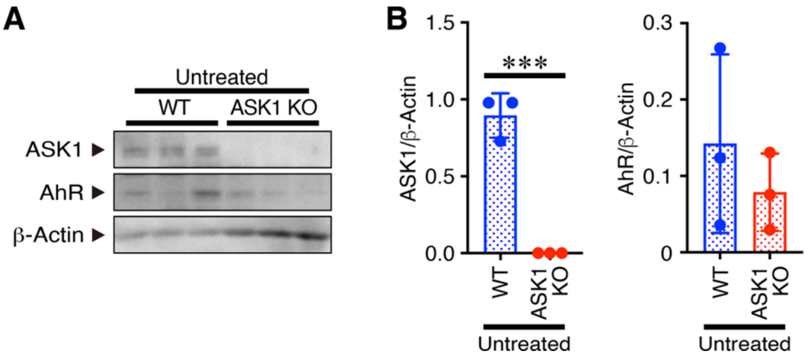

Supplementary Fig. S1. No obvious difference in AhR expression in the skin between WT and ASK1 KO mice under steady-state conditions. Cell lysates were prepared from the back skin of untreated WT and ASK1 KO mice (n = 3) subjected to Western blot analysis using anti-ASK1 and anti-AhR. Representative images are shown (A). Individual band intensities were measured using ImageJ software and normalized to β-actin (B). Data are shown as the mean ± SD. *P* values were determined using unpaired, two-tailed Student's *t*-test. ***$P <$ 0.001. Full-length blots are shown in Fig. S5A.

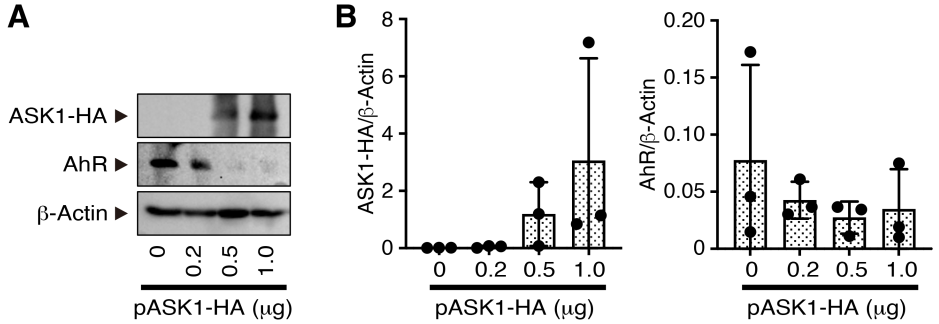

Supplementary Fig. S2. ASK1 overexpression trends to reduce the expression of endogenous AhR in the mouse keratinocyte cell line PAM212. PAM212 cells were transfected with increasing amounts of an HA-tagged ASK1 expression vector. Forty-eight hours later, cell lysates were prepared and subjected to Western blot analysis using anti-HA and anti-AhR antibodies, with anti-β-actin serving as a loading control. Representative images are shown in (A). Band intensities were quantified using ImageJ software and normalized to β-actin (B). Data are presented as the mean ± SD (n = 3). *P* values were determined using one-way analysis of variance with Dunnett's multiple comparisons test. Although the difference was not statistically significant, a trend toward reduced endogenous AhR expression was observed with ASK1 overexpression. Full-length blots are shown in Fig. S5B.

Fig. 3A

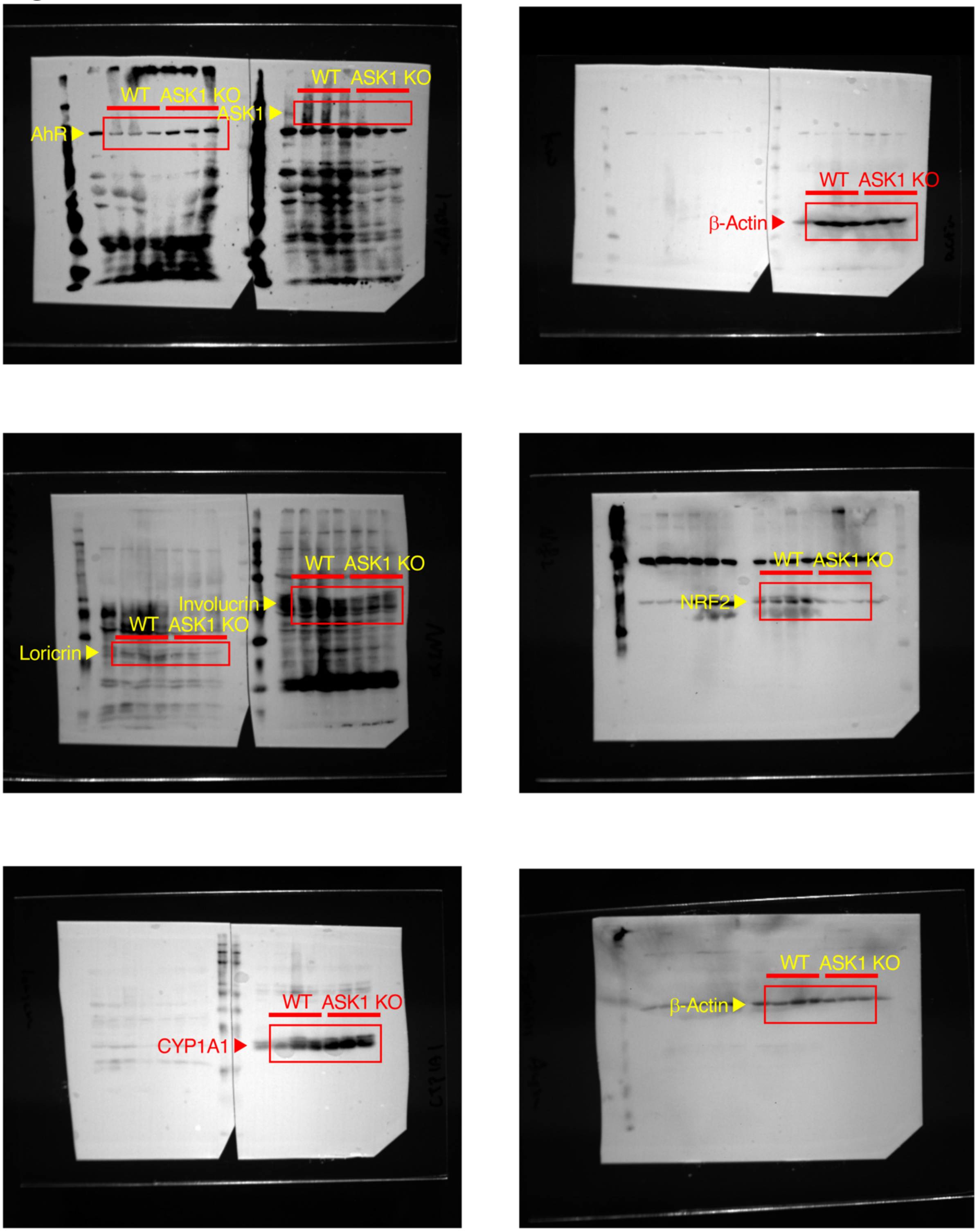

Supplementary Fig. S3. Full-length blots of Fig. 3A.

A Fig. 5A, B

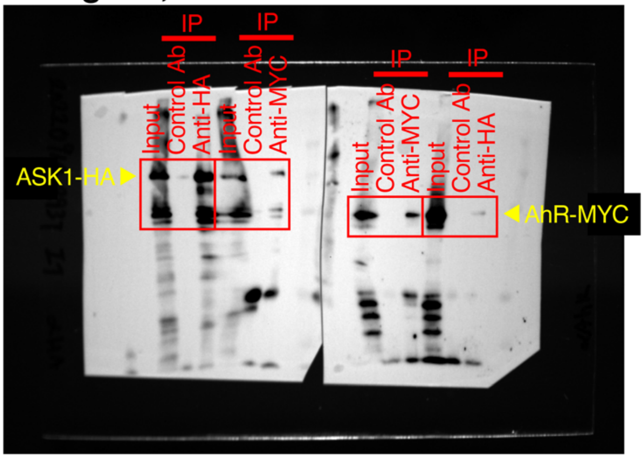

B Fig. 5C

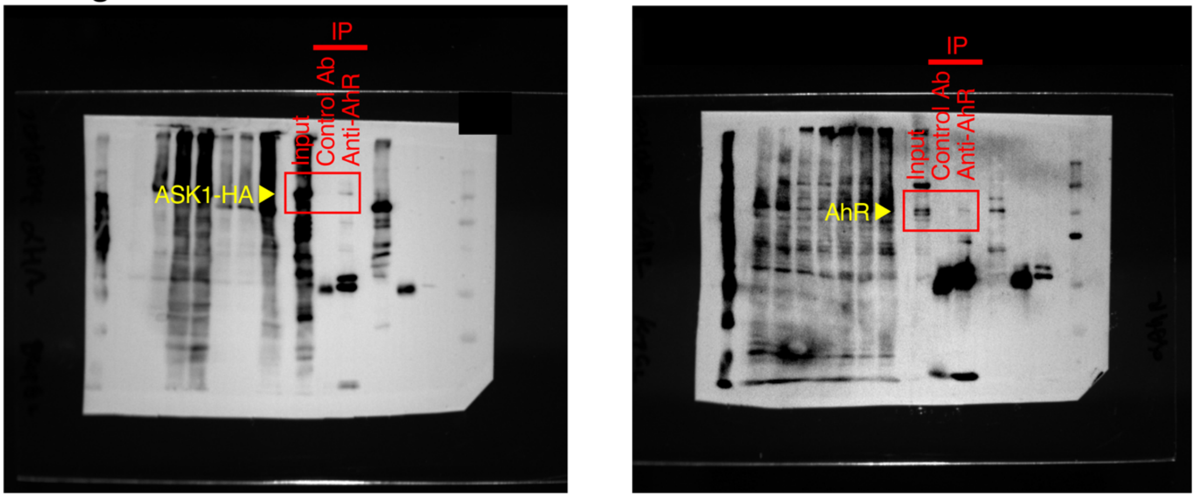

Supplementary Fig. S4. Full-length blots of Fig. 5A, B (A) and Fig. 5C (B).

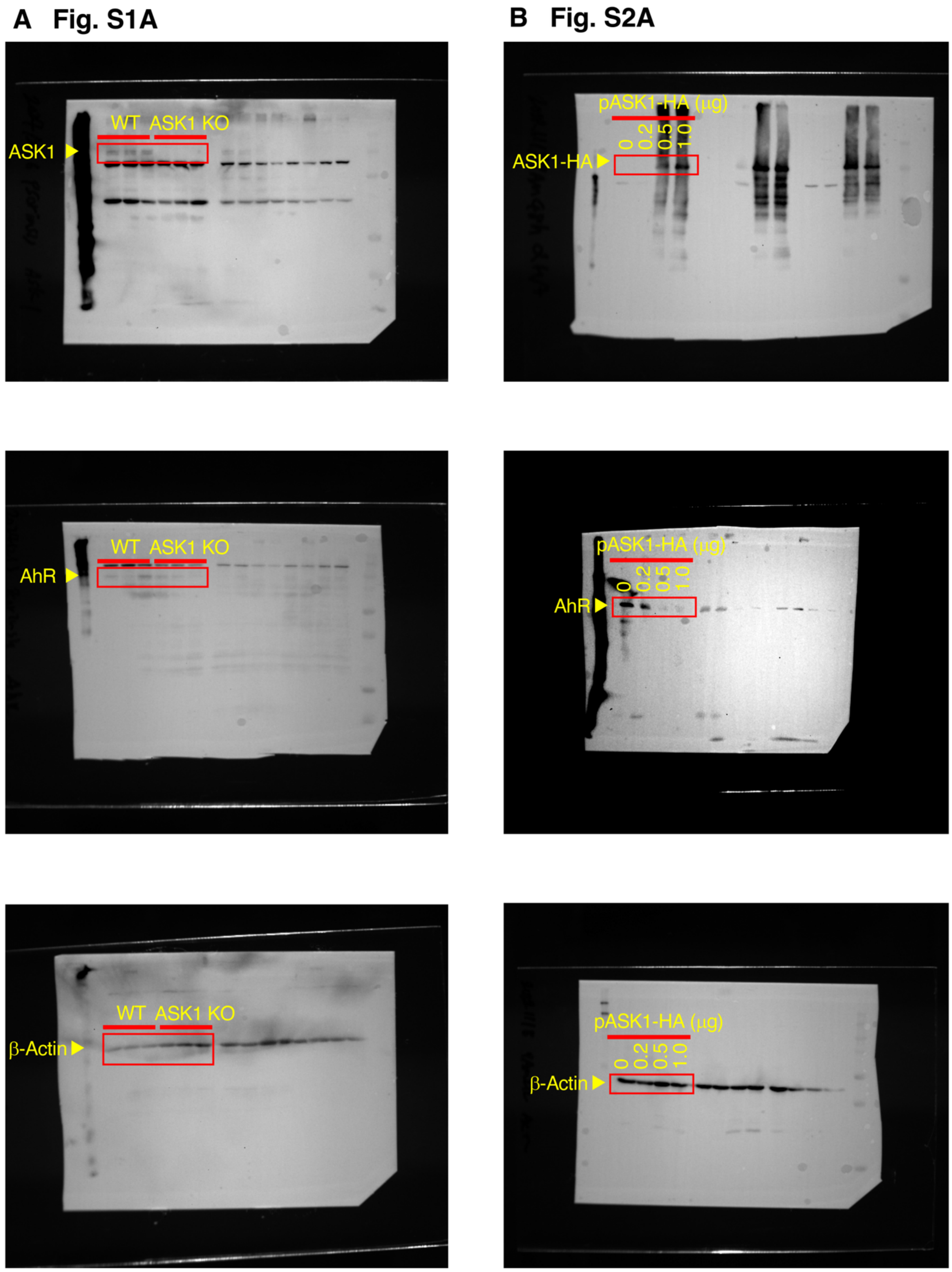

Supplementary Fig. S5. Full-length blots of Fig. S1A (A) and Fig.S2A (B).
